# Role of perivascular adipose tissue in endothelial dysfunction of adipose triglyceride lipase-deficient mice

**DOI:** 10.1186/2050-6511-13-S1-A18

**Published:** 2012-09-17

**Authors:** Karoline Pail, Sarah Winkler, Gerald Wölkart, Günter Hämmerle, Rudolf Zechner, Alois Lametschwandtner, Bernd Mayer, Astrid Schrammel

**Affiliations:** 1 Department of Pharmacology and Toxicology, Institute of Pharmaceutical Sciences, University of Graz, 8010 Graz, Austria; 2 Institute of Molecular Biosciences, University of Graz, 8010 Graz, Austria; 3 Division of Zoology and Functional Anatomy, Vessel and Muscle Research Unit, University of Salzburg, 5020 Salzburg, Austria

## Background

Perivascular adipose tissue (PVAT) has been recognized as an important factor in vascular biology due to its ability to produce a variety of vasoactive substances. In addition, it is regarded as an important source of proinflammatory mediators and reactive oxygen species (ROS). Experiments from our laboratory demonstrated that mice lacking adipose triglyceride lipase (ATGL), a crucial enzyme of triglyceride catabolism, suffer from severe micro- and macrovascular endothelial dysfunction. Since blood vessels of ATGL knockout mice (ATGL(−/−) mice) are surrounded by large amounts of PVAT, we investigated its potential contribution to the observed endothelial dysfunction.

## Methods and results

PVAT encompassing thoracic aortas of wild-type (WT) and ATGL(−/−) mice was isolated, characterized, and analyzed for protein and mRNA expression of different adipokines, inflammation markers, and sources of oxidative stress using real-time PCR and Western blot analysis, respectively. Knockout of ATGL caused a 7-fold increase in PVAT wet weight. While mRNA expression of adiponectin was reduced to about 50%, leptin mRNA was increased about 4-fold in ATGL deficiency. Adipose mRNA levels of the inflammation markers tumor necrosis factor alpha (TNF-α), monocyte chemoattractant protein 1 (MCP-1), and interleukin-6 (IL-6) were about 5-fold higher in ATGL-deficient PVAT. In addition, the NOX2/p67^phox^ complex was significantly upregulated at protein level. Heme oxygenase-1, which has been described protective against oxidative and inflammatory stress, was increased about 5-fold in ATGL deficiency. To distinguish between direct PVAT-mediated effects and those originating from the cardiac dysfunctional phenotype of the animals, we additionally analyzed tissue isolated from ATGL(−/−) mice with cardiomyocyte-specific overexpression of ATGL (rescued cardiac phenotype). Interestingly, the effect of ATGL knockout on TNF-α and leptin expression was reversible. By contrast, increased adipose NOX2/p67^phox^, MCP-1 and IL-6 expression persisted even upon restoration of cardiac function.

## Conclusions

Our data indicate that PVAT-derived inflammatory and NADPH oxidase-mediated oxidative stress might contribute to endothelial dysfunction in ATGL deficiency. The functional consequences of these findings are currently being investigated in our laboratory.

